# Survey to evaluate care of complex clients in residential setting

**DOI:** 10.1192/bjo.2021.577

**Published:** 2021-06-18

**Authors:** Rosa Sadraei, Puru Pathy, Michael Collins

**Affiliations:** 1 Nottinghamshire Healthcare NHS Foundation Trust; 2 Nottinghamshie Healthcare NHS Trust

## Abstract

**Aims:**

Delivering a new efficient assessment and shorter term secondary mental Health intervention service for individual sectors

**Background:**

In November 2015, there was a transition to services with the focus on delivering more efficient service to clients

Previously we had been a combined sector Service. This transition, a reduction in resources and a move away from delivering care Through specialist mental health teams created from the national service framework - such as Assertive outreach, early intervention in psychosis and community rehabilitation - to a more Streamlined generic service, catering for these differing groups of people using a “Pathways Model” approach

**Result:**

Across the two sectors we had 47 clients on CPA Pathway living in 24 hour residential Settings who all had a current care coordinator.

These 47 clients represented the workload currently of 2.8 FTE Band 6 care coordinators.

There were at Origin, 13 Residential/Nursing/Secure 24 Hour care providers, where clients were residing.

However of these 90% of residents lived in one of 5 settings, 3 settings in Ashfield and 2 in Mansfield.

Over 50% of individuals residing did not have existing connections with Mansfield or Ashfield before being placed into the area.

18 Clients (%38) were under section of the mental health act and 1client (%2) was on a life-Licence from criminal justice.

**Conclusion:**

Transfer of CPA Care Coordination Protocol

To send paper referral to our Single Point of Access Meeting at the listed address at the earliest point relocation/placement is confirmed.Formal handover meeting for care will be coordinated, not sooner than 3 months after the placement commences. It will be expected that services currently involved in provision of service continue to hold care responsibility in the interim period.

As we move to a paperless environment, provision of electronic documentation such has previous CPA documents, Risk assessments, social circumstance reports & Discharge summaries, would be greatly appreciated

